# Direct targeting of TDP-43, from small molecules to biologics: the therapeutic landscape

**DOI:** 10.1039/d1cb00110h

**Published:** 2021-06-21

**Authors:** Liberty Francois-Moutal, David Donald Scott, May Khanna

**Affiliations:** Department of Pharmacology, College of Medicine, University of Arizona 1501 North Campbell Drive, P.O. Box 245050 Tucson AZ 85724 USA maykhanna@email.arizona.edu +520-626-2204 +520-626-2147; Center of Innovation in Brain Science Tucson AZ 85721 USA; Bio5 Institute, University of Arizona Tucson USA

## Abstract

Tar DNA binding (TDP)-43 proteinopathy, typically described as cytoplasmic accumulation of highly modified and misfolded TDP-43 molecules, is characteristic of several neurodegenerative diseases such as Amyotrophic Lateral Sclerosis (ALS) and limbic-predominant age-related TDP-43 encephalopathy (LATE). TDP-43 proposed proteinopathies include homeostatic imbalance between nuclear and cytoplasmic localization, aggregation of ubiquitinated and hyper-phosphorylated TDP-43, and an increase in protein truncation of cytoplasmic TDP-43. Given the therapeutic interest of targeting TDP-43, this review focuses on the current landscape of strategies, ranging from biologics to small molecules, that directly target TDP-43. Antibodies, peptides and compounds have been designed or found to recognize specific TDP-43 sequences but alleviate TDP-43 toxicity through different mechanisms. While two antibodies described here were able to induce degradation of pathological TDP-43, the peptides and small molecules were primarily designed to reduce aggregation of TDP-43. Furthermore, we discuss promising emerging therapeutic targets.

## Abstract 2

Given the therapeutic interest of targeting TDP-43, this review focuses on the current landscape of strategies, ranging from biologics to small molecules, that directly target TDP-43. Regions targeted are shown on the 3D structure of RNA-bound TDP-43.

## Graphical abstract

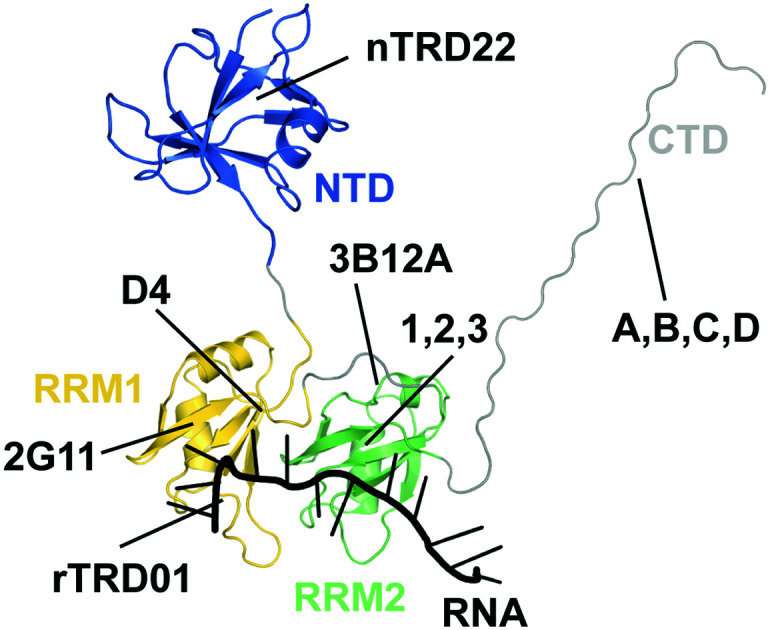

## Introduction

Tar DNA binding protein (TDP)-43 is a nucleic acid binding protein consisting of three domains, a folded N-terminal domain, two RNA Recognition Motifs (RRM1 and 2) and a mostly unstructured C-terminal domain where most of the known disease-associated mutations occur (reviewed in ref. 1). Mostly localized to the nucleus, TDP-43 is a ribonucleic acid-binding protein of the hnRNP family,^2^ that can translocate to the cytoplasm and mitochondria.^3–5^ As a nucleic acid binding protein, TDP-43 regulates RNA processing, including mRNA splicing-mainly by binding to UG-rich intronic regions-, RNA stability and transport (reviewed in ref. 6 and 7).

TDP-43 proteinopathies are usually characterized by cytoplasmic accumulation of misfolded and heavily modified TDP-43, accompanied by nuclear clearance.^8–10^ The origin of these aggregates is debatable, but stress granule and dynamics are thought to play a role.^11–13^ Even though the exact mechanisms remain largely unknown, pathological TDP-43 is thought to exert a plethora of deleterious effects ranging from nuclear transport inhibition by sequestering transport factors,^14^ to impaired autophagy and mitochondrial dysfunction,^15^ among others. The accumulation of those insults is thought to ultimately lead to neurite loss and subsequent neuronal death.

TDP-43 proteinopathies (review in ref. 16) include Amyotrophic Lateral Sclerosis – Frontotemporal lobar degeneration (ALS-FTLD) spectrum, facial onset sensory and motor neuronopathy (FOSMN), Guam Parkinson-dementia complex (G-PDC) with ALS (G-ALS), multisystem proteinopathy (MSP) and Perry disease. Limbic-predominant age-related TDP-43 encephalopathy (LATE) as well as cerebral age-related TDP-43 with sclerosis (CARTS), recently discovered in older patients (≥85 years old), have also been characterized as TDP-43 proteinopathies. Moreover, Alzheimer's disease and chronic traumatic encephalopathy (CTE) have exhibited concomitant TDP-43 pathology.

Given the interest in targeting TDP-43 in neurodegenerative diseases for therapeutic development, this report focuses on the existing strategies, ranging from biologics to small molecules, able to directly recognize or modulate TDP-43 pathology. Structural insights on targeted regions of TDP-43 will aid our understanding and may instruct future targeting. A more general overview of compounds has been recently written that describes how to mitigate pathological characteristics of TDP-43 such as expression or mislocalization.^17,18^

## Biologics

### Antibodies targeting TDP-43

**Fig. 1:**
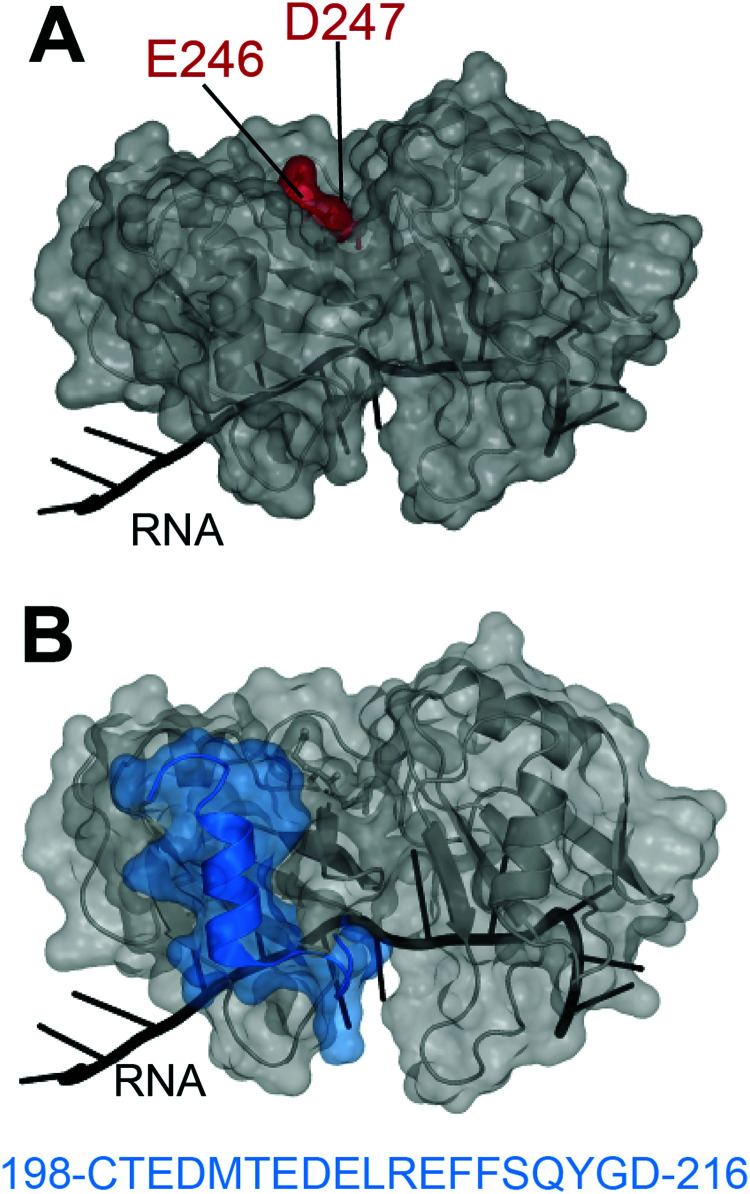
Targeting of TDP-43 by antibodies. (A) The 3B12A antibody is able to recognize aberrantly exposed E246 and D247 (red) in misfolded TDP-43 found in ALS tissues.^23^ (B) Two other antibodies, 2G11 and 2H1, developed by ref. 31 to recognize a TDP-43 sequence specific to humans (blue) and were able to recognize aggregates in samples from ALS-FTLD and LATE patients (PDB ID: 4bs2^54^).

In 2012, Shodai and collaborators developed an antibody against misfolded TDP-43 based on the assumption that RRM2 aberrant conformation is linked to pathological TDP-43.^19^ They specifically focused their efforts on residues E246 and D247 (Fig. 1A). Intriguingly, these residues are mostly buried within the natively folded TDP-43 structure but they are (i) part of an essential cleavage site in FTLD-U brains,^10^ (ii) localized in a defined amyloidogenic core^19,20^ and (iii) implicated in dimerization of RRM2 which is thought to lead to TDP-43 aggregation,^21^ similar to the dimer interface residues of SOD1 observed in familial ALS.^22^ Shodai and colleagues used a subconstruct of TDP-43 consisting of amino acids 241–260, to generate hybridomas which were later selected for their ability to bind TDP-43 RRM2 but not E246G/D247G RRM2. The resulting antibody was called 3B12A and recognized only highly dense TDP-43 structures in the nucleus, cytoplasmic TDP-43 with an impaired NLS as well as nearly 80% of TDP-43 inclusions in anterior horn cells of ALS patients. This led to the conclusion that exposed E246/D247 residues on TDP-43 may be a marker of TDP-43 inclusions in ALS/FTLD.

The same group later discovered the ability of the 3B12A antibody to induce proteasome degradation *via* a RIDPEDGETK sequence found in the 3B12A heavy chain.^23^ That sequence exhibits a high PEST score, which typically defines sequences rich in proline, glutamic acid, serine, and threonine that act as degradation signals.^24,25^ Further addition of a CMA (chaperone-mediated autophagy) sequence to the 3B12A antibody, resulted in efficient cell clearance of misfolded TDP-43 and reversed TDP-43 induced toxicity in HEK293A cells overexpressing cytoplasmic misfolded TDP-43 (TDP-43^mNLS,C173S/C175S^) as well as in cerebral cortex of newborn mice transduced with TDP-43^mNLS,C173S/C175S^.^23^ Even though further testing of this strategy needs to be considered, especially in adult models and/or patient derived induced stem cells, this was the first example of direct targeting of TDP-43 by an antibody leading to cellular clearance of pathological aggregates with therapeutic promise.

Another antibody was developed by Pozzi *et al.*,^26^ and is described as targeting the TDP-43 RRM1 domain. The rationale for this approach was based on TDP-43 RRM1 being implicated in aggregation *via* its misfolding,^27^ similar to RRM2, and/or by its oxidation.^28^ Another reason to target TDP-43-RRM1 was to disrupt its interaction with p65 NF-κB, which binds TDP-43 NTD and RRM1 and leads to inflammation in glial cells and sensitizes neurons to toxic insults.^29^ Although it should be noted that the binding between TDP-43 and p65 was shown through immunoprecipitation and not direct biophysical interactions. To generate hybridomas, they used TDP-43 encompassing part of the N-terminal region, the nuclear localization signal (NLS), and the entire RRM1 domain, which presumably spans amino acids (aa) 31 to 176 as this is the interface with p65 they describe.^29^ Then, they selected antibodies based on their ability to detect the input TDP-43 protein and to disrupt the p65 interaction, which led to the E6 antibody. Transfection of E6-derived vectors encoding single-chain fragment variable (scFv) antibodies induced proteasome and autophagy-driven degradation of TDP-43 in HEK293 cells. Viral-mediated expression of a scFv antibody (named VH7Vk9), further reduced LPS-induced inflammation markers in mouse cortex. Finally, treatment with VH7Vk9 improved cognitive function in TDP-43^G348C^ mice, a well-known model of familial ALS/FTD^30^ as well as motor performance in another model of familial ALS/FTD, namely TDP-43^A315T^ mice.^30^

Even though the exact binding site of the antibody remains obscure and could very well implicate TDP-43-NTD, VH7Vk9's ability to induce TDP-43 degradation by promoting polyubiquitination of TDP-43 without addition of a degradation signal is striking. Analysis of the VH7Vk9 sequence might reveal a PEST sequence, similar to ref. 23. Alternatively, the possibility that VH7Vk9 disrupts aggregates leading to TDP-43 being accessible to degradation was speculated by the authors.

Very recently, Trejo-Lopez developed two novel antibodies against TDP-43 RRM2, specific to the human form.^31^ These antibodies were raised against residues 198–216 of RRM2 (Fig. 1B), chosen based on the weaker conservation of this sequence between human and mouse (68%) compared to the rest of the sequence (97%). Antibodies 2G11 and 2H1 efficiently recognized human TDP-43 over the mouse version of the protein and were able to detect TDP-43 aggregates of ALS-FTLD and LATE, in a comparable manner to a commercially available anti-TDP-43 antibody. The authors describe that the novelty of these antibodies resides in their preference for cytoplasmic inclusions of TDP-43 over nuclear TDP-43, reminiscent of the anti-pSer409/410, which recognizes phosphorylated-TDP43. Structural analysis of the TDP-43 epitope recognized by 2G11 and 2H1 (Fig. 1B) reveals a sequence encompassing a loop and alpha-helix, with no known modifications or mutations attributable to disease state^1^ and this sequence is not at the interface with nucleic acids. The recognition of this sequence by the antibodies in cytoplasmic inclusions only, raises interesting questions. It would be fascinating to investigate the antibodies on normal patient samples to ascertain whether the site of recognition does indeed contain yet undiscovered PTMs that might not be detected by traditional mass spectrometry techniques.

In addition to the discovery of possible therapeutic strategies or diagnostic tools, these antibodies have uncovered unique surfaces on TDP-43, that may be differentially exposed in the disease state and relevant in TDP-43 cytoplasmic pathology.

### Peptides targeting TDP-43

In 2013, Liu and collaborators developed peptides targeting TDP-43-CTD to decrease TDP-43 aggregation and reduce subsequent toxicity in cells.^32^ The assumption was that TDP-43 aggregation occurs through self-interaction and TDP-43 derived peptides could mitigate aggregation. They defined these peptides using a peptide array, where overlapping TDP-43 peptides were tiled and incubated with full-length TDP-43. The interaction peptides, named A, B, C, D and E, were all sequences from the C-terminus region of TDP-43, but their exact sequences were not disclosed. All five peptides were able to reduce TDP-43 interaction with the peptide array, compared to a scrambled version of peptide C. Tat conjugated peptides inhibited TDP-43 aggregation of overexpressed TDP-43 as well as arsenite-induced TDP-43 inclusions in HeLa cells. The peptides were non cytotoxic but were not able to rescue TDP-43 related toxicity either. The authors hence concluded that inhibition of TDP-43 aggregation was not sufficient in itself to rescue subsequent toxicity.

**Fig. 2:**
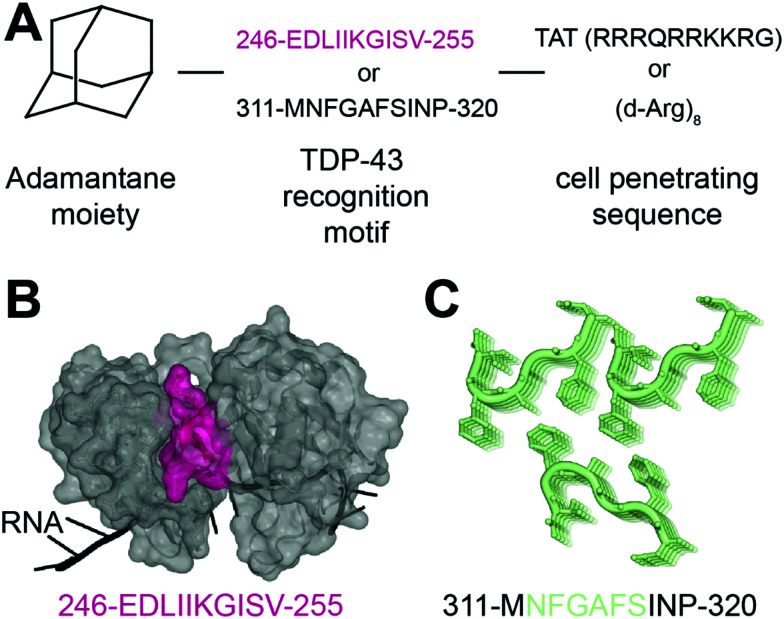
Peptides targeting TDP-43. (A) Structure of the peptides used in ref. 33. (B) The RRM2 sequence used for peptide D4 (pink) is mapped on TDP-43 structure (PDB ID: 4bs2^54^). (C) The sequence MNFGAFSINP is known to form aggregates (PDB ID: 5whn^59^).

In another recent effort to promote clearance of TDP-43 aggregates, Gao *et al.* designed several multifunctional peptides comprising a combination of three parts^33^ (Fig. 2A). The first part is a hydrophobic motif consisting of adamantane, a tricyclodecane cage-shaped compound. Adamantane-based hydrophobic tagging has been used to mimic the unfolded state of the target protein leading to its efficient degradation,^34,35^ and has the advantage of increasing blood–brain–barrier penetration of conjugated molecules.^36,37^ The second part is a TDP-43 “recognition motif” consisting of TDP-43 peptides, either 311-MNFGAFSINP-320 or 246-EDLIIKGISV-255. While the 246–255 sequence has been heavily described as an amyloidogenic core,^19,20^ the sequence 311–320 is less studied but was still reported as aggregation prone.^20^ It spans part of the GaroS1 region (aa. 273–317), that contributes to the formation of hydrogels^38^ and the very beginning of the hydrophobic region (residues 318–340) that can adopt both helical and thioflavin-T-positive filaments reminiscent of β-amyloid.^39–42^ The third part is a cell-penetrating peptide: Tat (RRRQRRKKRG) or Poly-d-Arginine (d-Arg)_8_. The C-terminal derived peptides were unsurprisingly not further pursued because of low solubility. The peptide D4, which contains E246/D247 residues, showed the strongest ability to reduce TDP-43 levels in an N2a cell line overexpressing TDP-43, while peptides lacking the hydrophobic adamantine moiety had no effect. The results were recapitulated in Drosophila overexpressing TDP-43. While enhanced degradation of TDP-43 by the D4 peptide is implied because of the adamantane moiety, the authors did not assess the effect of the D4 peptide on TDP-43 ability to autoregulate its own RNA or on cytoplasmic inclusions. Nevertheless, this study highlights again the seemingly central role of E246/D247 in TDP-43 misfolding and aggregation, making it an attractive therapeutic target in neurodegeneration.

## Small molecules

There are very few studies that focus on directly targeting TDP-43, probably due to the lack of pockets traditionally considered as druggable. The very first study targeting TDP-43, in 2013, used a high-throughput alpha-screen to find inhibitors of DNA binding.^43^ The emerging aminoquinoline compounds augmented caspase-7 degradation of TDP-43 in human neuroglioma H4 although the exact binding site and mechanism of action of those compounds were never elucidated.

**Fig. 3:**
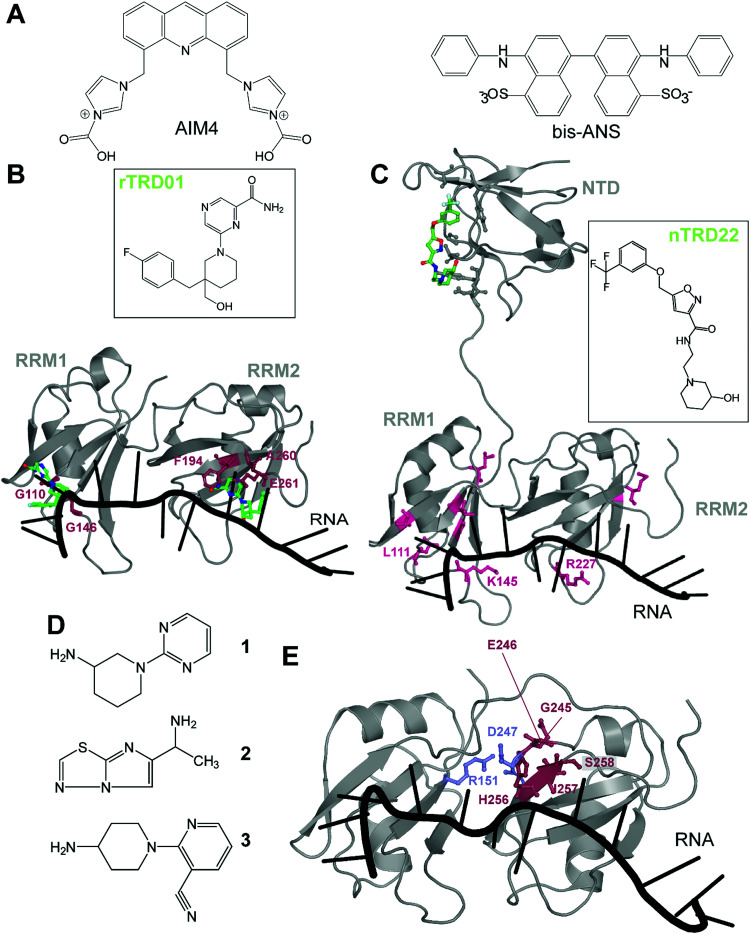
Small molecules targeting TDP-43. (A) Planar molecules able to bind TDP-43 CTD and mitigate LLPS formation or CTD driven aggregation. (B) rTRD01 (green, stick and balls representation) is thought to be able to bind both RRM domains of TDP-43 and displace nucleic acid binding (PDB ID: 4bs2).^54^ Residues shifted by rTRD01 binding to TDP-43 in HSQC-NMR are displayed in pink, stick and balls representation. rTRD01 is thought to bind RRM1 and RRM2. (C) Composite figure of TDP-43 NTD (PDB ID: 5mdi^63^) with TDP-43 RRM domains (PDB ID: 4bs2^54^). nTRD22 (green, stick and balls representation) binds to TDP-43 NTD and allosterically modulates residues implicated in nucleic acid binding (pink, stick and balls representation) to disrupt the binding. (D) Fragment compounds found to bind TDP-43.^53^ (E) Fragments are able to bind a pocket in TDP-43 RRM2 domain (red, stick and balls representation). The pocket is near the residues D247 and R151 (blue, stick and balls), shown to form a salt bridge, necessary for nucleic acid binding (PDB ID: 4bs2^54^).

In 2016, Prasad and collaborators^44^ examined the effect of acridine derivatives potential to mitigate TDP-43 aggregation, since acridine-based compounds were previously shown to inhibit amyloid formation of PrP^Sc^, among others^45^ (Fig. 3A). The authors thus report AIM4, [4,5-bis{(*N*-carboxy methyl imidazolium)methyl}acridine] dibromide, able to significantly reduce *in vitro* TDP-43 amyloid-like aggregation as well as TDP-43 inclusions in a yeast model cell. More recently, Babinchak *et al.* showed the ability of 4,4′-dianilino-1,1′-binaphthyl-5,5′-disulfonic acid (bis-ANS), and other similar compounds such as Congo Red, to modulate the liquid–liquid phase separation (LLPS) of TDP-43.^46^ While those studies demonstrate the potential of planar molecules in binding and mitigating TDP-43 aggregation/liquid phase separation, those compounds will require extensive validation in terms of toxicity and off-targeting properties since most of them are related to intercalant molecules or are known to be extremely toxic.

In recent years, our laboratory has focused on rational, structure-based targeting of TDP-43. Our first discovery used an *in silico* approach targeting the RRMs,^47^ more specifically the nucleic acid binding interface of RRM1, since mutations inhibiting nucleic acid binding completely reversed ALS phenotypes in TDP-43 overexpressed Drosophila models.^48^ We validated one compound, rTRD01, through NMR spectroscopy and Microscale Thermophoresis (MST), obtaining a binding constant in the micromolar range (*k*_D_ = 89.4 ± 0.8 μM), a relatively weak affinity that will be improved using structure activity relationship (SAR) studies. Interestingly, this compound was able to displace (GGGGCC)_4_ RNA, but not the native binding of (UG)_6_ repeats, probably due to lower affinity of TDP-43 for (GGGGCC)_4_ (nanomolar) compared to the canonical sequence (sub-nanomolar). Lastly, investigation of this compound in an ALS *Drosophila* model resulted in reduced locomotor defects.

These experiments characterized the first small-molecule directly targeting TDP-43 to identify therapeutics for ALS, even though RNA-recognition pockets are more shallow than conventional kinase ATP-binding pockets. It is important to note that although rTRD01 was obtained from a screen against RRM1, it could potentially also bind to RRM2 (Fig. 3B). HSQC-NMR shifts were observed in both RRM domains and it was not clear if this was due to allostery or direct binding to RRM2; the simplest explanation is direct binding. Further experiments will be used to determine if rTRD01 modulates these RRM domains independently. Our findings also display selective binding of TDP-43 to different RNA sequences. Interference with binding of specific RNA sequences could provide a therapeutic advantage by not altering native functions of TDP-43, as well as the possible identification of pathological biomarkers in neurodegenerative disease.

Another molecule we recently discovered is nTRD22 (Fig. 3C), found using the same pipeline as previously described, but targeting the N-terminal domain (NTD).^49^ The NTD is implicated in oligomer formation and support of nucleotide binding,^50–52^ among other functions. Interestingly, nTRD22 was shown to bind specifically to the NTD, with an affinity of 145 ± 3 μM, but was also observed to (i) shift residues in the RRM domains, as demonstrated by HSQC-NMR and (ii) displace canonical UG_6_ binding, unlike rTRD01 that directly targeted the RNA binding domains. We hypothesized that nTRD22 could affect the RRM domain through allostery, although current studies are ongoing to define how the NTD portion of TDP-43 influences the RRM domains. nTRD22 was further able to reduce TDP-43 protein levels in rodent primary motor neurons, recapitulating TDP-43 RNA binding-deficient mutants and supporting impaired TDP-43 binding to nucleic acids. Finally, nTRD22 mitigated motor impairment in a Drosophila model of ALS. This study not only shows the possibility of TDP-43 NTD to be targeted by small molecules, but also a possible therapeutic interest for such a strategy.

Through these works, potential allosteric interactions between TDP-43 domains were brought to light, even though further investigation is needed to understand the precise mechanism. This insight proposes new intrinsic regulatory roles of TDP-43, as well as a new landscape of therapeutics. As stated before, targeting the NTD resulted in a better inhibition of canonical UG_6_ RNA binding which might help achieve higher specificity with fewer off-target effects than rTRD01.

Other than our studies, an effort to find molecules targeting TDP-43 using a fragment-based NMR study identified hits that bound to the RRM domain.^53^ Three different NMR techniques were used, saturation transfer difference (STD), water ligand observed *via* gradient spectroscopy (WaterLOGSY) and Carr–Purcell–Meiboom–Gill (CPMG), to screen 89 cocktails, each containing 10 small molecules. A secondary screen on individual compounds, using the same NMR techniques, revealed four hits that were further examined using Chemical Shift Perturbations (CSPs) of the ^15^N-labeled tandem RRMs of TDP-43. Three of these hits, 1, 2 and 3, were able to induce significant CSPs on RRM2 residues G245, E246, H256, I257 and S258 (Fig. 3D and E). This group further validated the binding of these 3 compounds to TDP-43 RRM2 only using HSQC. Hit 1 was docked on TDP-43-RRM (PDB ID: 4bs2^54^) using the data driven approach HADDOCK and was predicted to form a hydrogen bond with the side chain of S258.

While the authors note that their hits 1–3 bind near a nucleic acid binding site on TDP-43 and that a fragment growth strategy can be used to target residues Asn259, and Glu261, implicated in RNA/DNA recognition, it should be noted that RRM2 binding to nucleic acid by itself is very weak, as previously shown by mutations in RRM2 domain,^21,55^ which questions the efficiency of this approach. An alternative strategy to inhibit nucleic acid binding could be to target D247, which makes a salt bridge with R151, stabilizing the RRM1-RRM2 orientation when RNA is bound^56^ (Fig. 3D). It is also interesting to note that the pathologically exposed and cleaved E246 is being targeted by compounds **1–3**. Given the role of E246/D247 in ALS, as described before, this avenue represents an interesting alternative therapeutic option.

## Conclusion

Given the attractiveness of targeting TDP-43 directly to treat neurodegenerative diseases, such as ALS-FTLD, the last few years have seen several different strategies emerge with variable success (Table 1). Antibodies, peptides and compounds have been designed or found to recognize specific TDP-43 sequences but alleviate TDP-43 toxicity through different mechanisms. While two antibodies described here were able to induce degradation of pathological TDP-43, the peptides and small molecules were primarily designed to target aggregation of TDP-43.

**Table 1:** Summary of candidate therapeutics directly targeting TDP-43

Name	Therapeutic type	Targeted site	Cell	Fly	Mice
3B12A	Antibody	Misfolded RRM2	Clearance of pathological TDP-43 in HEK293A cells	N/A	Clearance of TDP-43 aggregates in newborn mice transduced with TDP-43^mNLS,C173S/C175S^
VH7Vk9	Antibody	Misfolded RRM1	Induced proteasome and autophagy-driven degradation of TDP-43 in HEK293 cells	N/A	Improved cognitive function in TDP-^43G348C^ mice
Liu “A, B, C, D, E”	Peptide	CTD	Reduced aggregation of TDP-43 in HeLa cells but not toxicity	N/A	N/A
Gao “D4”	Peptide	CTD	Reduced TDP-43 levels in an N2a cell line overexpressing TDP-43	Recapitulated in Drosophila overexpressing TDP-43	N/A
AIM4	Small molecule	CTD	Reduced TDP-43 inclusions in a yeast *S. cerevisiae* model	N/A	N/A
rTRD01	Small molecule	RRM1/2	N/A	ALS Drosophila model results in reduced locomotor defects	N/A
nTRD22	Small molecule	NTD	Reduced TDP-43 protein levels in rodent primary motor neurons	Mitigated motor impairment in Drosophila model of ALS	N/A

Despite its lack of traditional targetable pockets, we and others have shown the ability of small molecules to bind TDP-43, albeit with a rather low affinity.^47,49,53^ SAR studies focusing on improving the affinity of existing compounds will reveal how effective these strategies are by increasing potency without increasing off-targeting. Alternatively, exploiting conjugation of proteolysis targeting chimera (PROTAC) to these small molecules is a potential new avenue. PROTAC relies on two covalently combined molecules, one recognizing the protein target and the other able to recruit an E3 ubiquitin ligase for ubiquitination and subsequent degradation of the protein target.^57^ Even though TDP-43 is already ubiquitinated in pathological aggregates, antibodies targeting TDP-43 were able to direct TDP-43 towards degradation with and without direct ubiquitination.^23,26^ PROTAC targeting of TDP-43 is thus a potential new strategy, with the caveat that the approach does not target well-folded physiological TDP-43 over inclusions. From this review, it is clear that targeting residues E246 and especially D247 is of high interest, since they seem to be exposed in TDP-43 inclusions.^23^ The coupling of targeting this pathological surface with a degradation strategy led to efficient clearing of aggregates.

In defining therapeutics that can target the predominantly unstructured C-terminus of TDP-43, there are two plausible modes of action: the peptides could either modulate or disrupt protein–protein (PPI) interactions that occur through the C-terminus. There are also other mechanisms we do not consider, such as modulation of condensate, either directly or through condensate formation, or binding to other non-proteinaceous entities. On the other hand, targeting the TDP-43 CTD seems highly challenging. Peptides targeting the CTD failed;^32,33^ it is highly unstructured in the soluble form and forms polymorphic fibrils,^58^ making rational design of molecules difficult. Further, there is disease and tissue heterogeneity of TDP-43 C-terminal fragments in terms of composition and structure. Nevertheless, recent efforts, led by the Eisenberg laboratory among others, in structurally characterizing TDP-43 CTD peptides,^42,58–61^ with and without mutations, will help tremendously in targeting this region. Based on similar efforts to structurally characterize tau segments driving aggregation by MicroED, inhibitors of tau aggregation were developed by targeting VQIINK steric zipper interfaces with peptides derived from the same sequence (VQIINK).^62^ Based on these studies, they suggest tau amyloid polymorphism with distinct structures of tau fibrils. Similar efforts on structurally defining patient derived TDP-43 inclusions, which are possibly polymorphic, are very likely to provide in the future the mean to design small molecules or biologics that might specifically target these structures.

Although none of the strategies described here have led to compounds that have reached clinical trials, several promising targeting options have been developed. Given the challenges consistently faced in neurodegenerative disease drug development, there needs to be extreme care in choosing appropriate: (i) animal models, potentially in combination with patient derived stem cells, to translate specific disease hallmarks being targeted, (ii) formulation, delivery route and ability to eventually cross the blood–brain–barrier by the drug candidates and (iii) better clinical trial design, for optimal and successful development of therapeutic candidates.

Further experiments are needed to define efficiency and/or utilize combinations of therapies to achieve clearance of toxic aggregates as well as inhibition of TDP-43 pathology.

## Conflicts of interest

There are no conflicts to declare.

## Supplementary Material
